# Lead Poison

**Published:** 1877-03

**Authors:** 


### LEAD POISON.

All who use water, conducted into their dwellings by leaden pipes, are interested in the question, whether the lead, under any circumstances, can impregnate the water with poison? It is certainly so. No argument, beyond that of often ascertained facts, is necessary to prove this. But the water delivered to one family will cause a slow, wasting, and fatal disease, while the water from the same sources, introduced into the next house, is used for years without any appreciable ill results. The simple reason is, that in the fatal case, *the flow of water is obstructed*, either by a too sudden bend in the pipe, or by a pebble or other indestructible impediment. *Standing water will corrode lead.* Simple dampness will corrode lead, and bring out its poisonous qualities. Obstructions to a flowing stream of water will arrest any particles in that water which are not the pure water itself: those particles are usually of vegetable origin; and as soon as a small portion of them are collected in any part of the pipe, or rather arrested by the obstacle, destructive decomposition begins; and the gases escaping in consequence of this process act upon the lead, and make it poisonous. Every man, therefore, who builds a home for himself, should understand that, if it is to be supplied with water by leaden pipes, his life, and that of all his, depends on the fidelity of the plumber; and as plumbers trust their work to apprentice boys, and uninterested journeymen, the owner should watch the laying of every foot of pipe, and not allow an inch of it to be put down during his absence; the points to which he should bend his most fixed attention are:

*First.* Let the pipe be laid as straight as possible.

*Second.* Let every joint be made perfectly smooth.

*Third.* See to it, that not an atom of anything be left inside the pipe which would obstruct the smallest particle of any substance, whether it be leaf, or wood, or grass, or hair, or string, or worm, or insect, or anything else.

Meanwhile, as long as it is certain that still water corrodes lead, the most unthinking person will draw the practical inference, that the water from the hydrant should be allowed to run off for the first five or ten seconds after turning the faucet, to get a supply for drinking or eating.

**CROUP** is an inflammation of the inner surface of the windpipe. Inflammation implies heat, and that heat must be subdued or the patient will inevitably die. If prompt efforts are made to cool the parts in case of an attack of croup, relief will be as prompt as it is surprising and delightful. All know that cold applied to a hot skin cools it, but all do not as well know and understand, that hot water applied to an inflamed skin will as certainly cool it off. Hence the application of ice-cold water with linen cloths, or of almost boiling water with woolen flannel, are very efficient in the cure of croup. Take two or three pieces of woolen flannel of two folds, large enough to cover the whole throat and upper part of the chest, put these in a pan of water as hot as the hand can bear, and keep it thus hot, by adding water from a boiling tea-kettle at hand; let two of the flannels be in the hot water all the time, and one on the throat all the time, with a dry flannel covering the wet one, so as to keep the steam in to some extent; the flannels should not be so wet, when put on, as to dribble the water, for it is important to keep the clothing as dry as possible, and the body and feet of the child comfortable and warm. As soon as one flannel gets a little cool, put on another hot one, with as little interval of exposure as possible, and keep up this process until the doctor comes, or until the phlegm is loose, the child easier, and begins to fall to sleep; then gently wrap a dry flannel over the wet one which is on, so as to cover it up thoroughly, and the child is saved. When it wakes up, both flannels will be dry. The same results will follow if cold water is used, the colder the better; the cloths should be of muslin or linen and of several folds thickness, large enough to cover the whole throat and the upper part of the breast.

